# A Life-Threatening Case of Severe Leptospirosis in Wales, UK: Recognising Severe Disease in Non-endemic Settings

**DOI:** 10.7759/cureus.98918

**Published:** 2025-12-10

**Authors:** Hosham Ahmed, Anas Ishqair, Rehan Khalil, Sala Fadelallah

**Affiliations:** 1 Medicine, University Hospital of Wales, Cardiff, GBR; 2 Internal Medicine, University Hospital of Wales, Cardiff, GBR

**Keywords:** acute kidney injury, dialysis, leptospirosis, severe, uk - united kingdom

## Abstract

Leptospirosis is an infectious disease that is uncommonly seen in the United Kingdom. It is typically associated with occupational exposure among sewage workers, farmers, veterinarians and abattoir employees. The illness is known by other terms such as seven-day fever, haemorrhagic fever and Weil’s disease. The prevalence of leptospirosis outside of the United Kingdom is well documented, however it remains relatively uncommon to be encountered in hospitals inside the UK. While the majority of cases encountered are mild, severe disease with jaundice and acute renal failure has been described, highlighting the importance of clinician awareness in non-endemic settings.

We summarise the case of a 54-year-old male with a two-week history of malaise, fevers and oliguria who was initially treated for sepsis of unknown origin. The patient rapidly deteriorated and was admitted to ITU for haemofiltration whilst further investigations took place to establish a diagnosis.

## Introduction

This case aims to highlight the potential severe and even fatal consequences of leptospirosis so that clinicians should be able to identify risk factors and investigate promptly should the suspicion be there even in non-endemic environments. 

Leptospirosis is a zoonotic infection which is caused by the spirochaete Leptospira interrogans. The organism thrives in warm, humid environments, making leptospirosis far more prevalent in tropical and subtropical regions [1]. However, the phenomenon of the global spread of leptospirosis has been increasingly recognised: incidence trends show that temperate and high-income countries are no longer excluded from risk, with travel, migration and changing recreational exposures contributing to this shift [2]. Surveillance within the European Union/European Economic Area shows that notification rates of leptospirosis increased by approximately 5% per year over 2010-2021, illustrating a rising burden even in temperate regions [3].

Clinically leptospirosis has a biphasic presentation - an early phase and a second immune phase. The early leptospiraemic phase generally lasts around a week and may be mild or even subclinical. Individuals may only have flu-like symptoms during this stage. Subconjunctival haemorrhage/redness is also commonly seen. The second phase can lead to severe infection known as Weil’s disease; this gives rise to renal failure, hepatitis and even aseptic meningitis [4]. A diagnosis in temperate regions such as the UK can be challenging given the wide array of symptoms and overlap with other infectious diseases such as dengue, malaria and influenza virus [5]. Transmission occurs primarily through exposure to water or soil contaminated with the urine of infected animals, particularly rodents. Rates of transmission are affected by changes in climate as well as one's environment; for example, we know the bacteria itself occupies warm conditions and tends to be found in rivers, sewers and waterlogged soil. Laboratory confirmation remains key in establishing a diagnosis. It is achieved through serological or molecular testing such as Leptospira-specific IgM or polymerase chain reaction (PCR).

## Case presentation

In this case we discuss a 54-year-old gentleman who presented to the acute medical unit of a tertiary hospital in Wales with a two-week history of nausea, fevers, myalgia, diarrhoea and poor urine output. He has a past medical history of asthma and osteoarthritis but is otherwise well in himself and independent of activities of daily living. A social history revealed that he did have a 10 pack-year smoking history as well as cannabis use and works as a recruitment manager as well as a gardener. There was no history of foreign travel, animal bites or new sexual partners. 

On arrival into the Medical Assessment Unit the patient was hypotensive and tachycardic. A high-grade fever of 39.4^o^C was also recorded. On examination there was evidence of jaundice; his abdomen was distended but soft. Chest auscultation revealed mild wheeze. A cardiovascular exam was normal. Given his presentation and haemodynamic instability the patient was treated for sepsis with broad-spectrum antibiotics, piperacillin-tazobactam and clarithromycin. The doses were 4.5g and 500mg respectively, both given twice daily. Despite fluid resuscitation his renal function had deteriorated, necessitating ITU admission for haemofiltration and invasive monitoring. The patient showed a good clinical response and was able to be weaned off vasopressors. Following his ITU stay he was then transferred to the nephrology unit for further dialysis sessions before eventually being discharged from hospital with a course of doxycycline and follow-up in the renal clinic. His renal function fluctuated over the next several months but never recovered to his baseline level. Fortunately he was not left with any other long-term complications. 

Investigations

Initial investigations revealed a high white cell count, neutrophilia, thrombocytopenia and significant hepatorenal failure. A trend of all blood test values is outlined below in Table 1. A venous blood gas showed a mildly raised lactate and normal pH.

**Table 1:** A trend of blood test markers over time ALP: alkaline phosphatase, ALT: alanine aminotransferase, eGFR: estimated glomerular filtration rate

Blood counts and biochemistry	Day 1	Day 5	Day 15 (discharge day)	Normal range
WBC	17.9 x10^9^/L	12.7 x10^9^/L	9.4 x10^9^/L	4-11 x10^9^/L
Platelets	112 x10^9^/L	84 x10^9^/L	302 x10^9^/L	150-400 x10^9^/L
Sodium	118 mmol/L	135 mmol/L	142 mmol/L	133-146 mmol/L
Potassium	3.4 mmol/L	4.1 mmol/L	4.4 mmol/L	3.5-5 mmol/L
Urea	18.1 mmol/L	9.2 mmol/L	22 mmol/L	2.5-7.8 mmol/L
Creatinine	590 mmol/L	168 mmol/L	435 mmol/L	58-110 mmol/L
eGFR	9 ml/min/1.73m^2^	37 ml/min/1.73m^2^	12 ml/min/1.73m^2^	>60 ml/min/1.73m^2^
C-reactive protein	390 mg/L	15 mg/L	5 mg/L	<5 mg/L
Creatine kinase	12447 U/L			40-320 U/L
Bilirubin	51 umol/L	207 umol/L	51 umol/L	<21 umol/L
ALP	166 U/L	125 U/L	120 U/L	30-150 U/L
ALT	86 U/L	121 U/L	74 U/L	<59 U/L
Albumin	22 g/L	26 g/L	34 g/L	35-50 g/L

**Figure 1:**
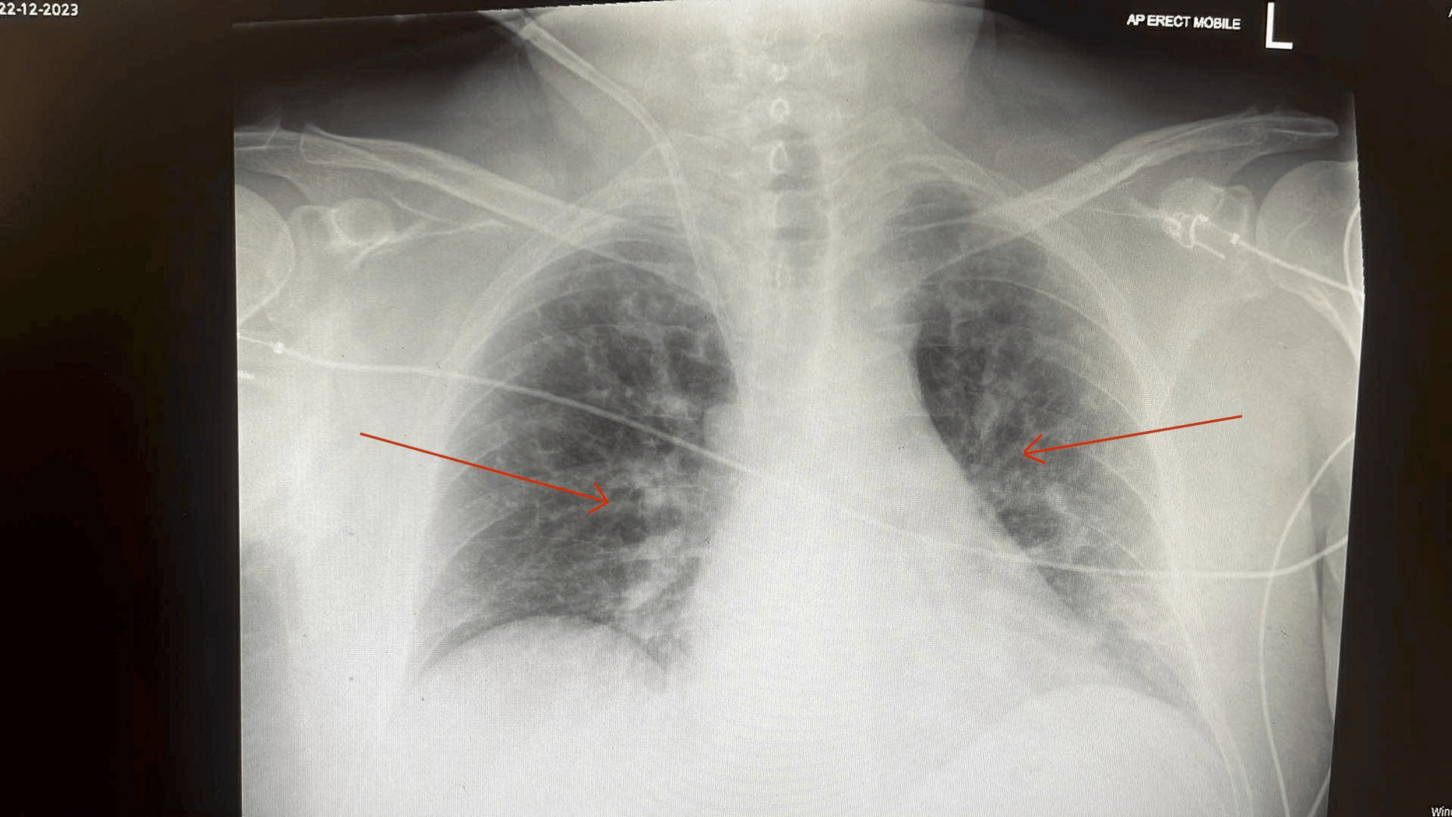
Anteroposterior Chest X-Ray Arrows highlighting increased lung markings suggestive of interstitial oedema

A chest radiograph (Figure 1) had shown bilateral opacification particularly in the mid and lower zones and increased perihilar markings. This was likely representing fluid overload in addition to a possible lower respiratory tract infection. An initial blood culture was negative after five days of incubation. A full vasculitis screen was done (which returned as negative) due to the acute presentation of the renal failure. An echocardiograph was done two days into admission which showed a small amount of biventricular dysfunction but no significant valvular pathology. Unfortunately, an ejection fraction was not able to be quantified. An extensive microbiology panel of investigations were done in order to identify a particular organism as outlined in Table 2. A blood PCR test returned positive within seven days as well as Leptospirosis IgM 12 days into admission.

**Table 2:** A table outlining microbiology investigations EBV: Epstein-Barr virus, CMV: cytomegalovirus, Ag: antigen, Ab: antibody

Bacteria	Result
Legionella	Negative
Leptospirosis IgM	Detected
Streptococcus pneumoniae	Negative
Virus:	
Adenovirus	Negative
Coronavirus	Negative
Influenza A and B PCR	Negative
Enterovirus PCR	Negative
Hepatitis C Antibody	Negative
Hepatitis B Surface Antigen	Negative
HIV Ag/Ab	Negative
EBV PCR	Negative
CMV PCR	Negative

Differential diagnosis

Similar to many infectious diseases, leptospirosis can mimic other viral and bacterial infections. Differential diagnoses included septic shock of unclear origin, viral hepatitis, acute interstitial nephritis, autoimmune or vasculitic disease such as antineutrophil cytoplasmic antibodies (ANCA)-associated vasculitis, or other tropical infections such as dengue or malaria.

In the absence of travel and with negative vasculitis and culture results, leptospirosis became the leading diagnosis once serology was confirmed.

## Discussion

Leptospirosis presents with a broad spectrum of clinical manifestations ranging from subclinical infection to severe, life-threatening illness. Even in individuals without obvious animal exposure, it remains a significant yet under-recognised cause of acute febrile illness in temperate regions, including the UK [4,6]. Recently, one recent European case described a 64‑year‑old male presenting with hyperbilirubinaemia, acute kidney injury and thrombocytopenia in the absence of travel to endemic zones - comparable to the presentation we describe here [7].

Renal involvement is a well-recognised complication of leptospirosis, often presenting as acute kidney injury (AKI). Importantly, a European case series demonstrates that AKI is a key predictor of severity in leptospirosis [8]. In this case, the patient developed renal failure requiring both haemofiltration and then subsequent dialysis consistent with the known pathophysiology of leptospiral nephropathy. The pathogenesis involves direct tubular damage by Leptospira and immune-mediated interstitial nephritis [9]. Contributing factors such as hypovolaemia and rhabdomyolysis, reflected by the markedly elevated creatine kinase in this case, further exacerbate renal injury. The diagnosis of leptospirosis in the UK can be challenging due to its relative rarity and nonspecific early symptoms. It often mimics viral or other bacterial infections, leading to delayed recognition. This case highlights the importance of obtaining a detailed occupational and environmental exposure history. Notably, recent contact with freshwater or animals (particularly rodents) should raise clinical suspicion, even in non-endemic regions. Liver dysfunction is another recognised manifestation. Although serum aminotransferase elevations are typically modest, hyperbilirubinaemia may be profound, as seen here. Similar findings were described by Cardoso et al. (2022) and in other European case reports, reinforcing that hepatic involvement does not necessarily imply fulminant hepatic necrosis but reflects cholestatic injury. As outlined earlier, laboratory confirmation typically involves serological testing (e.g., microscopic agglutination test) or PCR in early stages. In our case, PCR was used, enabling prompt initiation of targeted antibiotic therapy, which is crucial in preventing progression to severe disease.

The patient's recovery underlines the effectiveness of early diagnosis and antimicrobial treatment, while indicating that the burden of the condition may be underestimated. Clinicians in the UK should maintain a high index of suspicion, especially during warmer months or following flooding events, which increase the risk of exposure.

## Conclusions

This case highlights that leptospirosis remains a diagnostic challenge due to its non-specific presentation and overlap with other infectious diseases. Our patient initially appeared to have sepsis of unclear origin, and only after extensive investigations was leptospirosis confirmed. By this time, he had already developed significant renal failure requiring haemofiltration and ongoing dialysis. His clinical course highlights not only the severity that leptospirosis can reach, but also the time frame in which recognition and prompt treatment can alter outcomes. In this scenario, specifically, it was within one week. This case also contributes to the limited but expanding body of UK-based literature on leptospirosis-induced renal failure, reinforcing the need for awareness and timely intervention to prevent adverse outcomes. What makes this case particularly interesting is the absence of recent travel or overt animal exposure, which might usually raise suspicion. His history as a gardener, however, likely brought him into contact with contaminated soil or water, showing how seemingly low-risk exposures can be relevant in temperate climates. For clinicians, the key lesson is to keep leptospirosis in mind when faced with severe febrile illness and organ dysfunction, even outside endemic areas. Timely diagnosis and supportive management can be life-saving, and greater awareness will improve recognition of this under-reported disease.
